# PRRS Monitoring by Processing Fluids on Italian Swine Breeding Farms

**DOI:** 10.3390/ani13121946

**Published:** 2023-06-10

**Authors:** Matteo Tonni, Claudia Romeo, Nicoletta Formenti, Maria Beatrice Boniotti, Flavia Guarneri, Livio Colosio, Simone Andreoni, Federico Scali, Giovanni Loris Alborali

**Affiliations:** 1 Istituto Zooprofilattico Sperimentale della Lombardia e dell’Emilia Romagna—IZSLER, Via Bianchi 9, 25124 Brescia, Italy; 2 Swine Technical Services, Boehringer Ingelheim Animal Health Italia S.p.A., Via Vezza D’Oglio 3, 20139 Milano, Italy

**Keywords:** PRRSV, processing fluids, herd classification, disease surveillance, pigs, porcine reproductive and respiratory syndrome

## Abstract

**Simple Summary:**

Processing fluids (PFs) are novel diagnostic specimens consisting of serosanguineous exudate obtained from tissues after piglets’ castration. PFs can be used for some diagnostic tests that would otherwise require a blood sample. This has the advantage of not subjecting piglets to an additional stressful procedure and, at the same time, reducing sampling costs. In the present study, the efficacy and reliability of a monitoring plan for porcine reproductive and respiratory syndrome (PRRS), one of the main pig diseases, based on PF sampling has been assessed in the Italian swine production system. Twenty-five breeding herds were monitored for 4–5 months by RT-PCR performed on both PFs and blood serum (the standard specimen used for monitoring). Based on the results obtained from each method, the herds were classified following the standard PRRS status categories. The two methods fully agreed in discriminating between herds with stable and unstable PRRSV circulation. However, we observed a slight discrepancy in classifying high- and low-prevalence herds within unstable herds. We conclude that PFs are a reliable material for the PRRSV surveillance and classification of breeding herds, but in case of unstable circulation, a strategy combining blood and PF sampling is recommended.

**Abstract:**

The porcine reproductive and respiratory syndrome (PRRS) control strategy within swine breeding farms is based on herd classification relative to PRRSV infection status. This study aims to assess the efficacy of a monitoring plan based on processing fluids (PFs) by comparing it with the classification of herds based on the analysis of blood serum. Twenty-five breeding herds were enrolled in the study, with at least five consecutive batches sampled from each herd. Each batch was tested for PRRSV by RT-PCR performed on (i) pre-weaning blood serum from 30 piglets and (ii) PFs from all the male piglets in the batch. PRRS categories following the Holtkamp classification were assigned based on the results of each testing protocol. The two protocols assigned the same category to 18 out of 25 herds: while they showed perfect agreement in identifying positive unstable and stable herds, we observed some discrepancy in discriminating between low- and high-prevalence classes within unstable herds. PFs are thus a reliable sample to assign PRRS categories in Italian breeding herds characterized by widespread PRRSV circulation. However, in case of an unstable epidemiological scenario, we recommend the adoption of an integrated monitoring strategy that combines blood sampling with PFs.

## 1. Introduction

Porcine reproductive and respiratory syndrome (PRRS) is one of the most important diseases affecting the swine industry due to its severe impact on pigs’ health [1] and relevant economic consequences in most pig production areas [2]. The virus responsible for PRRS (i.e., PRRSV) induces a poor immune response in pigs and is characterized by high genetic heterogeneity and long persistence within the host population [3,4,5,6]. The circulation of PRRS is affected by many factors (herd management, pig flows, environmental features, etc.) [7]. Both the virus’s traits and its epidemiology make disease control very challenging [8].

The first step in the control strategy within breeding farms is herd classification based on infection status. Currently, the most commonly applied classification is based on virus shedding and previous exposure to the virus [9] defined by testing a representative subpopulation of pigs. Such a PRRS classification system proposed by Holtkamp and colleagues has been modified over time [10], up to the current classification of herds into five categories [9]: positive unstable high prevalence (I-A, <75% of weaning batches test negative by reverse-transcription polymerase chain reaction, RT-PCR, after 90 days of testing on pools), positive unstable low prevalence (I-B, >75% of weaning batches test negative by RT-PCR after 90 days of testing on pools), positive stable and positive stable with vaccination (II and II-vx, 100% of weaning batches test negative to wild type strains by RT-PCR after 90 days of testing on pools), provisional negative (III, 100% of naïve breeding animals kept in the herd for at least 60 days—or their litters—test negative by ELISA), and negative (IV, 100% of adult breeding animals—or their litters—test negative by ELISA). The diagnostic evidence requirements list several possible diagnostic materials including blood serum and processing fluids (PFs) [9].

The reliability of PFs as a testing material has been highlighted by several studies [11,12,13,14], that have shown how RT-PCR on PFs can even achieve greater sensitivity compared to RT-PCR on blood sera because more individuals are included in the testing pools. Moreover, since PFs are a waste material readily available following routine castration, their use for PRRSV diagnosis avoids additional, ad hoc blood sampling, sparing further stress to piglets and saving time for workers. For these reasons, many Italian farms have turned to monitoring programs which include PF samples. Nevertheless, although Holtkamp’s classification [9] of breeding herds is currently applied, and monitoring based on PFs is implemented, there is no evidence of the effectiveness of this method in correctly assigning PRRS categories in Italian swine farming. The prevalence of PRRSV in Italy has been estimated at around 90% [7], and the greatest effort by the swine industry is thus focused on controlling positive unstable and stable farms.

This study is aimed at assessing the reliability of a PF-based monitoring program by comparing the PRRS classification of breeding herds based on the laboratory outcome obtained from the analysis of blood serum and PFs.

## 2. Materials and Methods

### 2.1. Study Population

Twenty-five commercial breeding farms located in the north of Italy were enrolled in this study. The study was performed between July 2019 and June 2020, according to farmer and farm veterinarian compliance. All of the farms followed a three-week batch management system. The herd size ranges from 250 to 1200 sows (median 950 sows). All of the enrolled herds adopted a PRRS vaccination protocol using a modified live vaccine administered only to sows every four months and no later than 3 weeks before parturition. A monthly PRRSV monitoring program based on RT-PCR analysis performed on blood serum collected from weaning-age pigs was already routinely adopted at all of the farms. No restriction based on the clinical history of recent or late PRRS outbreaks was applied.

### 2.2. Sampling and Diagnostic Testing

Since all of the farms adopted a three-week batch management system, an every-three-weeks monitoring plan was set up by farm veterinarians. The monitoring plan was carried out through two simultaneous sampling protocols. The first protocol (hereafter “PFs protocol”) consisted of collecting PFs from the testicles of all male piglets following routine castration, according to the method proposed by Lopez and colleagues [11]. Since routine tail docking is forbidden in Italy, tails were not collected. The second protocol (hereafter “blood protocol”) consisted of sampling blood from 30 randomly-selected pre-weaning pigs, according to the protocol proposed by Holtkamp et al. [9]. When more than one farrowing room was present, the sample was balanced across them. Each batch of pigs was therefore tested with both protocols, at 3–4 days of age by PFs and before weaning (at approximately 28 days) by blood samples.

All samples were submitted by farmers and/or farm veterinarians to Istituto Zooprofilattico Sperimentale della Lombardia e dell’Emilia Romagna (IZSLER) and tested for PRRSV using a Virotype PRRSV RT-PCR kit (Indical Bioscience, Leipzig, Germany). The positive cut-off cycle-threshold (Ct) value was set at ≤37. The PFs were pooled by 10–15 litters (each pool including PFs from approximately 70–100 piglets), while blood serum was pooled by five piglets. Each batch was defined as positive when at least one of the pools tested was found to be positive.

### 2.3. Data Analysis

To assign a herd to a PRRSV category, at least five consecutive batches (range: 5–9) were tested with both protocols, meaning that each herd was monitored for at least 100 days. Following [9], a heard was assigned to category I-A (positive unstable, high prevalence) when less than 75% of the batches tested negative, category I-B (positive unstable, low prevalence) when more than 75% of the batches tested negative, and category II (positive stable) when 100% of the batches tested negative.

The agreement between the two testing protocols in defining the PRRS status was estimated using the caret package [15] in R Statistical Software (v6.0.92; [16]). The accuracy and its 95% confidence interval, Cohen’s kappa, and sensitivity and specificity of processing fluids relative to serum were calculated.

## 3. Results

### PRRSV Status of the Herds

**Figure 1:**
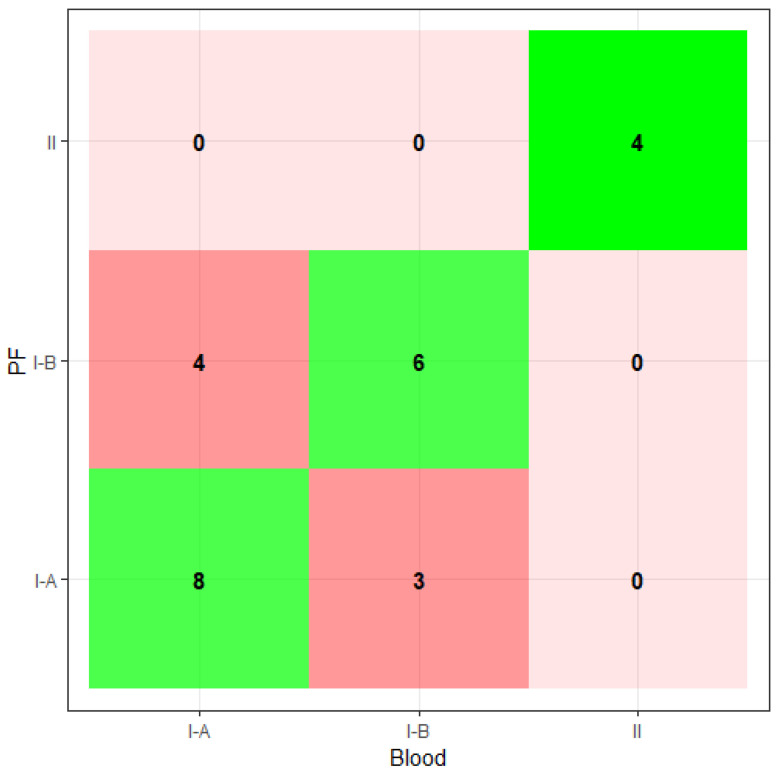
Heatmap chart showing the occurrence of agreement (green) and disagreement (red) between the processing fluids testing protocol and the blood testing protocol in defining PRRSV categories [9] in breeding pig herds (n = 25).

Overall, PRRSV was detected by PFs in 42 batches and by blood in 43 batches out of the 157 batches tested. In all cases, the strains belonged to PRRSV-1. The two testing protocols assigned the same PRRSV category to 18 out of 25 herds (72% accuracy; 95%CI: 51–88%; *p* = 0.013) with Cohen’s k = 0.52, indicating moderate agreement (see Table 1 for detailed data). In four cases (16%), the blood serum protocol assigned the herds to category I-A, while they were assigned to category I-B based on PFs (Figure 1). The reverse occurred in three cases (12%). The sensitivity of processing fluids relative to serum in assigning categories I-A and I-B was 67% (with intra-class specificity of 77% and 75%, respectively). However, the two testing protocols always agreed in discriminating between unstable (I-A or I-B) and stable (II) herds, with both identifying the same four herds as stable and the other 21 as unstable (100% accuracy; 95%CI: 86–100%; Cohen’s k = 1).

## 4. Discussion

This study showed that the PFs protocol is effective at assigning a herd to PRRSV categories [9] even in Italian breeding herds, generally characterized by smaller sizes than US herds where this sampling was validated.

Since Italy is characterized by widespread PRRSV circulation with a high proportion of positive farms [7], the main goal in the Italian epidemiological context lies in the discrimination between stable (II or II-vx) or unstable (I-A and I-B) herds. Our data showed that the blood serum and PFs protocols were in complete agreement in discriminating between unstable and stable herds. From a management point of view, a monitoring program based on PFs could be effective when applied in the Italian context.

However, agreement between the two protocols was lower when discriminating between the I-A (high prevalence) and I-B (low prevalence) categories. The PFs protocol assigned four herds to I-B instead of the I-A category by detecting a smaller proportion of positive batches compared to the blood sampled at the pre-weaning stage. Conversely, in three cases, the reverse occurred and the PFs identified a higher proportion of positive batches than blood, assigning the herd to the high-prevalence category. Of course, this discrepancy occurs because the two materials are sampled at two different time points in piglets’ life stages, thus capturing two diverse epidemiological pictures. The PFs protocol will indeed miss PRRSV in herds where piglets become infected during the second or third week of life, while by relying exclusively on blood sampled at three weeks of age, piglets that were positive at birth but have no detectable viremia at the pre-weaning stage might be missed. Since PRRSV viremia is known to change over time and the purpose of monitoring programs is to identify at least one positive sample per sampling event, combining PFs and blood sampling protocols would allow to increase the sensitivity of the monitoring program. This strategy has been suggested by Trevisan and colleagues [13], especially on farms that are in the virus elimination stage.

Definitively, while PFs showed good reliability for PRRSV monitoring in Italian breeding farms, the application of both PFs and blood serum protocols is still recommended [9,13]. Holtkamp and colleagues suggest as well to apply a monitoring scheme that includes PFs within a program that also involves blood serum collection [9]. More in detail, PFs alone are considered an effective diagnostic material to promote a herd from I-A to the I-B category and to maintain it in the I-B, III, and IV categories. However, to promote to or to maintain a herd in category II, concurrent testing on blood serum is required, while PFs are not considered a valid sample to promote a herd to category III or IV. The farms enrolled in this study had a previous PRRSV classification status and none of them were eligible for a category upgrade. The application of the PFs protocol was thus enough to define the herd classification. As previously mentioned, PFs are very useful in times of epidemiological stability because they achieve higher herd-level sensitivity compared to blood sampling [12]. It is important, however, that they are combined with blood serum when samples test continuously negative for several weeks or when the viremia trend is changing [9].

The PRRSV monitoring plans adopted in this study included at least five consecutive batches, which means approximately four to five months of surveillance, a tight timeframe that needs to be considered as a limitation of the study. Further studies monitoring a longer timeframe and including a greater number of herds with a more unstable PRRS epidemiology would be needed. Finally, it must be noted that in the European Union (EU), processing procedures are considered a poor practice for animal welfare [17] and methods alternative to surgical castration are under consideration. As a consequence, the collection of PFs in EU member states could no longer be an option in the future, and studying new protocols for effective PRRS monitoring should be a priority.

## 5. Conclusions

Our results showed that PFs are a reliable material for PRRSV surveillance and the related classification of breeding herds in high-circulation epidemiological contexts such as Italian swine production. Our findings are consistent with previous studies [11,12,13] and lead us to recommend the use of PFs, preferably combined with blood serum, on farms with active PRRSV circulation.

## Figures and Tables

**Table 1:** PRRSV categories (as defined in [9]) assigned to each of the 25 breeding pig herds based on processing fluids and blood testing protocols.

Herd ID	PRSSV Category		Agreement ^1^
	Processing Fluids	Blood Serum	
1	I-B	I-A	N
2	I-B	I-A	N
3	I-A	I-A	Y
4	I-A	I-B	N
5	I-B	I-B	Y
6	I-B	I-B	Y
7	I-A	I-A	Y
8	I-A	I-B	N
9	II	II	Y
10	I-B	I-A	N
11	I-B	I-B	Y
12	I-A	I-A	Y
13	I-B	I-A	N
14	I-A	I-A	Y
15	I-B	I-B	Y
16	I-B	I-B	Y
17	I-B	I-B	Y
18	I-A	I-A	Y
19	I-A	I-B	N
20	II	II	Y
21	II	II	Y
22	I-A	I-A	Y
23	I-A	I-A	Y
24	I-A	I-A	Y
25	II	II	Y

^1^ Y: the testing protocols assigned the same PRRSV category; N: the testing protocols did not assign the same PRRSV category.

## Data Availability

Raw data supporting the conclusions of this article will be made available by the authors upon request, without undue reservation.
